# Deformation Monitoring Based on SBAS-InSAR and Leveling Measurement: A Case Study of the Jing-Mi Diversion Canal in China

**DOI:** 10.3390/s24123871

**Published:** 2024-06-14

**Authors:** Pengjun Luo, Xinxin Jin, Ding Nie, Youzhi Liu, Yilun Wei

**Affiliations:** 1 State Key Laboratory of Simulation and Regulation of Water Cycle in River Basin, China Institute of Water Resources and Hydropower Research, Beijing 100038, China; luopengjun233@gmail.com; 2 China Institute of Water Resources and Hydropower Research, Beijing 100038, China; jxxkl@sina.cn (X.J.); nieding@iwhr.com (D.N.); weiyilun@edu.iwhr.com (Y.W.)

**Keywords:** InSAR, subsidence, accuracy verification, canal

## Abstract

The Jing-Mi Diversion Canal is a large-scale water diversion project in Beijing. Routine monitoring is crucial for the reliability and stability of urban water supply. Compared with traditional monitoring methods, interferometric synthetic aperture radar (InSAR) has the advantages of large scale and high accuracy. Based on the small baseline subset InSAR, 187 ascending and 102 descending SAR images obtained from Sentinel-1 were used to detect the deformation along the diversion canal from 2017 to 2023. The results show that there was a sinking trend along the diversion canal. The subsidence was serious in the first half of the canal, and continued to sink from 2019 to 2020. The subsidence was alleviated in 2023. Combined with leveling measurements, the InSAR deformation monitoring results of important pumping station buildings were verified. The measurement accuracy of InSAR can reach the millimeter level. We extracted the groundwater level time series and subsidence for risky canal segments. Through pixel-by-pixel comparison, it was found that fluctuations in groundwater level would have some impact on surface deformation. Severe local subsidence or uplift deformation occasionally occurred. To ensure the safety of water diversion, the monitoring and maintenance of relevant pump station buildings in risky areas should be increased in the future.

## 1. Introduction

As a worldwide geological environmental disaster, land subsidence is usually caused by changes in the natural environment and by human intervention [1]. Subsidence may cause infrastructure damage or failure and permanent geological deformation [2]. In water diversion projects, the subsidence of soil will seriously endanger project safety, normally through pipeline deformation or rupture [3], leakage or collapse caused by lining cracks [4], or increased flood risk due to subsidence [5]. To ensure the safe operation of water diversion projects, regular deformation monitoring is required. At present, the widely used method is leveling measurement, or, in some areas, global navigation satellite system (GNSS) measurement is used [6,7,8]. Leveling and GNSS measurements have high accuracy, but they are only suitable for discrete points and cover a sparse space, making it difficult to conduct large-scale observations along the canal route.

Interferometric synthetic aperture radar (InSAR) is a space detection technology developed in the late 1960s. It has the advantages of large scale, high temporal resolution, millimeter-level accuracy, rapidity, and economy [9,10]. Massonnet et al. [11] first used two synthetic aperture radar (SAR) images for differential processing (i.e., DInSAR) to observe the deformation caused by an earthquake, which has prompted DInSAR to be widely used in landslide interpretation, surface deformation monitoring and other fields [12,13]. However, traditional DInSAR has certain limitations in distinguishing displacement and atmospheric effects and is strongly affected by temporal and geometric decorrelation. To eliminate these effects, multi-temporal InSAR (MT-InSAR) has been developed. Ferretti et al. [14] used ESA data and stable natural reflectors or permanent scatterers for interferometry (i.e., PS-InSAR), achieving millimeter-level terrain motion detection. Berardino et al. [15] proposed the small baseline subset differential interference technology (i.e., SBAS-InSAR), which improved sampling frequency and spatial coverage of the monitoring area. Mora et al. [16] optimized a small baseline subset and proposed a time evolution solution for detecting and tracking the small-scale deformation phenomena, which made it possible to use InSAR to monitor small areas. Nowadays, MT-InSAR has been increasingly developed in the field of deformation monitoring and has been gradually applied to landslides [17,18,19], high-rise buildings [20], airports [21], roads and bridges [22], etc.

In hydraulic engineering, InSAR has been used to monitor dam deformation and reservoir landslides [23,24], but its application in water diversion engineering is limited. Han et al. [25] measured the spatiotemporal changes of subsidence around all American canals, confirming the practicality of InSAR in canal subsidence. Miller et al. [26] used L-band UAVSAR and Sentinel-1 data to measure the subsidence along the California aqueduct and identified dangerous areas. Tapete et al. [27] used PS-InSAR to monitor the local deformation of Roman aqueducts and verified its accuracy using GPS. The most common method for verifying the accuracy of InSAR technology was to compare it with other satellites or GNSS. However, this approach often yielded unsatisfactory results. This is because the observation angle and flight direction of different satellites are different, and the deformation direction of the features in the monitored area is often unknown. Consequently, what is obtained by the InSAR technology is only a projection of the real deformation in the direction of the line of sight of the radar (LOS) [28]. Many researchers approach this by ignoring horizontal deformation and then projecting the LOS deformation in the vertical direction. However, horizontal deformation cannot be ignored in some engineering projects, such as pumping stations, dams, and other hydraulic engineering projects that are driven by water flow. In addition, most of the research on InSAR deformation monitoring only emphasizes measurement of the deformation that has already occurred, without further analyzing the impact of external factors on the deformation, which is unfavorable for future maintenance work. In summary, using reasonable methods for accuracy verification and exploring factors that affect deformation can fill the research gap in related fields, which is conducive to ensuring the safe operation of hydraulic engineering.

In this study, we design a monitoring process for a diversion canal, as can be seen in Section 2. In the first section of Section 3, we gain an overall understanding of the deformation patterns along the diversion channel, and then conduct an annual analysis of the unstable areas. This is beneficial for a comprehensive understanding of the monitoring area. In the second section, the relationship between groundwater level and subsidence is analyzed. This is useful to further investigate the causes of surface deformation. Finally, detailed monitoring and accuracy verification are conducted in the third section. This is important for the application of this method in engineering practice.

## 2. Materials and Methods

### 2.1. Research Area

**Figure 1:**
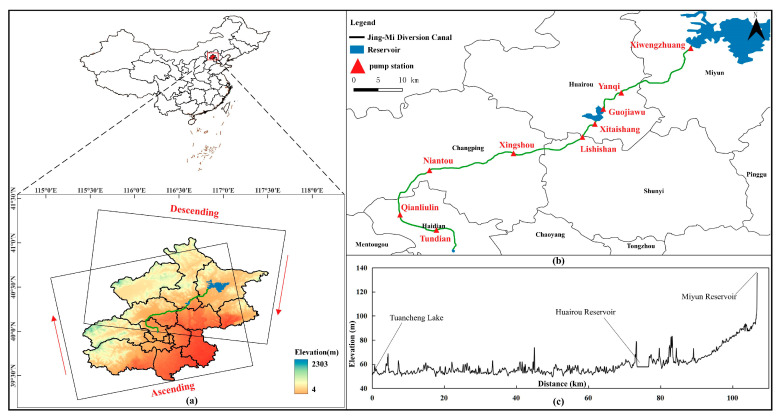
Overview of the research area. (**a**) Location and SAR images coverage range. The arrow points in the direction of satellite flight. (**b**) The route of Jing-Mi Diversion Canal. (**c**) The elevation distribution along the route.

The Jing-Mi Diversion Canal (JMDC) is a large-scale water diversion project that diverts water from the Miyun Reservoir to the Beijing metropolitan area. It started its year-round water diversion in 1989, with a total length of about 103 km. The Miyun Reserve Regulation and Storage Project (MRRSP) is a supporting facility of JMDC, and includes a nine-stage booster pump station and a 22-km pipeline, as shown in Figure 1. It plays an important role in solving the problem of the mismatch between the water supply of the South to North Water Diversion Project and the water consumption in Beijing. The overall layout of the project is to take water from Tuancheng Lake and divert it in the opposite direction through the JMDC. After passing through six pumping stations (Tundian, Qianliulin, Niantou, Xingshou, Lishishan and Xitaishang), the water is pressurized and diverted to Huairou Reservoir. After Huairou Reservoir returns the water to the source, it is pressurized and sent to Miyun Reservoir through a three-stage pumping station (Guojiawu, Yanqi, Xiwengzhuang). Among these, the water transfer scale from Tuancheng Lake to Huairou Reservoir reaches 20 m^3^/s, and the water transfer scale from Huairou Reservoir to Miyun Reservoir reaches 10 m^3^/s.

To ensure the safe operation of the project, the China Institute of Water Resources and Hydropower Research began installing monitoring equipment on buildings such as pumping stations, gates, and pipelines in December 2015. They mainly used once-a-day automated monitoring for seepage pressure and groundwater level. Subsidence and horizontal deformation were monitored once a month. The subsidence observation work was strictly carried out in accordance with the specifications for the second order level in China. The closure error measured per kilometer is within 2 mm. The horizontal deformation observation adopts laser projection, which has a centering error of 3 mm. However, manual measurement is time-consuming and labor-intensive, and it is difficult to carry out large-scale continuous observation. It is of great significance to achieve automated monitoring as soon as possible.

### 2.2. Data

**Figure 2:**
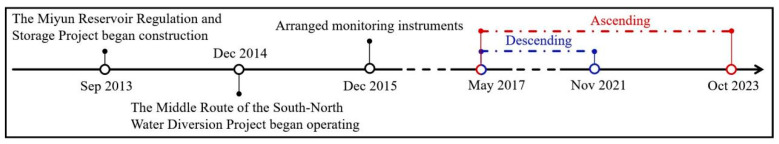
Timeline for supporting facilities construction and SAR images acquisition.

Sentinel-1 consists of two polar orbiting satellites, Sentinel-1A and Sentinel-1B. They operate alternately day and night. The satellite performs C-band synthetic aperture radar imaging. The collection interval for SAR images is mostly 12 days. Sentinel-1 requires an uplink track to cover the entire JMDC route. Single look complex (SLC) data obtained by Sentinel-1 in interferometric wide swath mode were used to monitor the diversion canal at a resolution of 5 m × 20 m (range × azimuth). In this study, we used 187 ascending SAR images from 20 May 2017, to 4 October 2023 to calculate the LOS deformation along the JMDC, with an azimuth angle of 347° and an incidence angle of 42°. One hundred two descending SAR images from 7 May 2017 to 30 November 2021 were used for auxiliary analysis, with an azimuth angle of 194° and an incidence angle of 37°. Then, leveling measurements were used to verify the accuracy of the subsidence values. The date and timeline of SAR images acquisition are shown in Figure 2. 

### 2.3. Method

**Figure 3:**
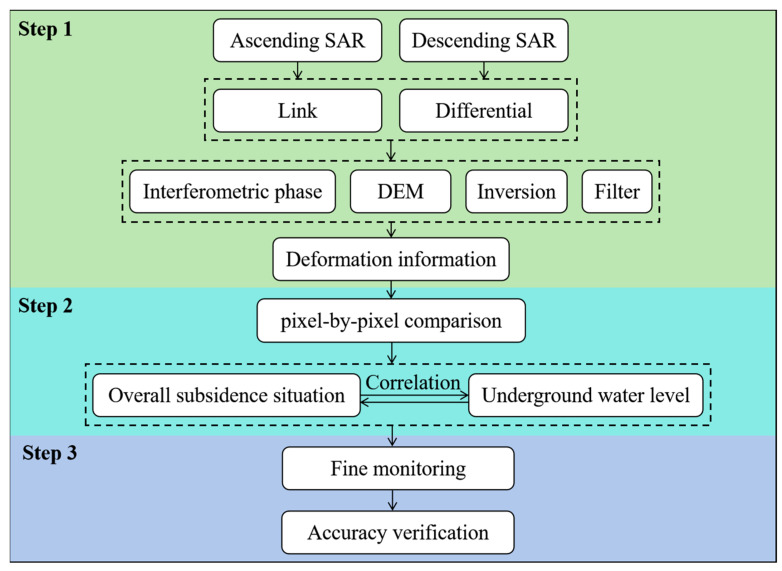
The flow chart of the deformation monitoring.

The combination detection of PSs and DSs may improve the accuracy of subsidence monitoring, but this depends on the PS points. In addition, PS-InSAR has requirements on the number of images that are too strict [29,30], meaning that the existing number of descending images is sometimes unable to meet its requirements. SBAS-InSAR can obtain more interferometric pairs with shorter temporal and spatial baselines than PS-InSAR by increasing the time sampling through small baseline combinations. To ensure that we could obtain satisfactory results, we decided to use SBAS-InSAR for the calculation and validate it with leveling results. We designed a deformation monitoring process for the diversion canal. It is divided into three modules in total, and the flow chart is shown in Figure 3. 

**Figure 4:**
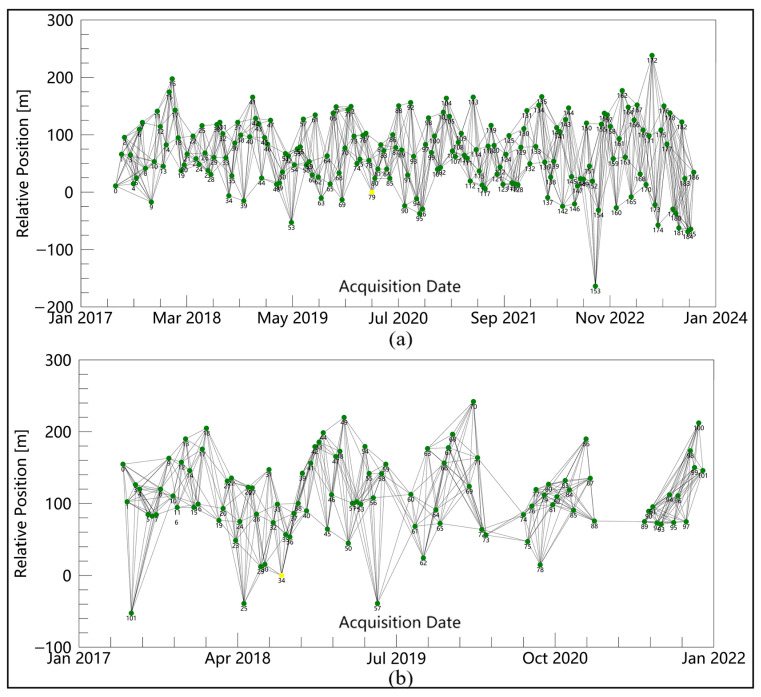
The temporal and spatial baseline of SAR images. (**a**) Ascending and (**b**) descending data. Yellow dots represent the master image, green dots represent the slave image.

The first step is to process remote sensing images. We obtain land deformation information by using small baseline subset InSAR. Firstly, one must link SAR images in time series. The maximum spatial baseline threshold percentage should be set to 45% and the maximum temporary baseline should be set to 180 days. The time–position plot of all ascending and descending SAR images is shown in Figure 4. Then, one should generate the interferometric phase through differential processing. The phase unwrapping method is minimum cost flow (MCF), with an unwrapping coherence threshold of 0.3. The expression for interferometric phase is as follows:

(1) $$\varphi_{Int}=\varphi_{Top}+\varphi_{Def}+\varphi_{Flat}+\varphi_{Atm}+\varphi_{Noi}$$

where $\varphi_{Top}$ is the topography phase, $\varphi_{Def}$ is the deformation phase, $\varphi_{Flat}$ is the flat phase caused by the flat earth effect, $\varphi_{Atm}$ is the atmosphere phase, and $\varphi_{Noi}$ is the noise phase. We use ALOS 12.5 m digital elevation model (DEM) as a reference elevation to eliminate the topography phase and use spatial filters to eliminate the atmosphere and noise phases. The filtering method is Goldstein, with a maximum alpha of 2.5 and a minimum alpha of 0.3. Then, we use Jacobi singular value decomposition inversion [31] to remove the flat phase and obtain time series deformation values. Finally, all processing results are projected to the WGS84 coordinate system.

The second step is to investigate the deformation situation along the entire diversion canal, identify unstable areas, and explore the impact of groundwater level on subsidence. Affected by groundwater exploitation, the surface deformation in Beijing is dominated by subsidence [32,33,34]. Due to the sensitivity of InSAR to vertical deformation when compared with horizontal deformation, it is usually assumed that horizontal deformation can be ignored when investigating large-scale deformation [35,36,37]. In view of the above, to quickly investigate the overall situation on a large scale, we project the calculated LOS deformation into the vertical direction as the subsidence, which can be expressed as follows:

(2) $$d_{S}=\frac{d_{LOS}}{\cos\alpha }$$

where $d_{S}$ is the subsidence value; $d_{LOS}$ is the deformation in LOS direction, with positive values indicating closer proximity to the satellite and negative values indicating further proximity; and α is the radar incident angle. In this process, we only use monthly ascending SAR images for calculation, in order to quickly investigate the subsidence situation. Afterwards, all ascending and descending images are input to analyze subsidence and horizontal deformation. We generate the vertical and horizontal deformation distribution map along the diversion canal. The negative values represent subsidence and westward horizontal deformation. The positive values represent uplift and eastward horizontal deformation. The mapping resolution is 15 m × 15 m. Finally, an annual analysis of the risk areas is performed based on the groundwater level.

**Figure 5:**
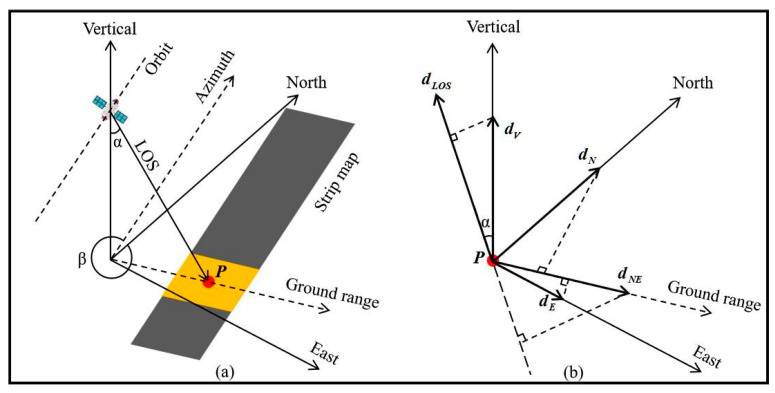
Three-dimensional decomposition of deformation. (**a**) SAR system relative to geographic coordinate system. (**b**) Decomposition of P-point deformation.

The third step is to conduct a fine monitoring of the supporting facilities of the diversion canal and verify the accuracy. We used the calculation results of rising SAR images for time series analysis. The LOS deformation is decomposed into three dimensions, including vertical, east–west, and north–south directions, as shown in Figure 5. The antenna footprint (orange block) moves along the satellite orbit. P is a point in the monitoring area. $d_{NE}$ is the projection of east–west and north–south deformation in the ground range direction. We do not disregard horizontal deformation but instead project all leveling measurement results onto the line-of-sight direction to obtain more reliable results.

As an illustrative example, the deformation monitoring time series results of InSAR are validated using the results of on-site leveling measurements. For the results of leveling measurement, they should be projected onto the LOS direction using the following equation [38]:

(3) $$d_{LOS}=d_{V}\cdot \cos\alpha -d_{E}\cdot \sin\alpha \cdot \cos\beta +d_{N}\cdot \sin\alpha \cdot \sin\beta$$

where $d_{V}$ is the vertical deformation, with upward being positive and downward being negative; $d_{E}$ is the east–west deformation, with the east being positive and the west being negative; $d_{N}$ is the north–south deformation, with the north being positive and the south being negative; and β is the azimuth angle. Finally, one should extract the InSAR results based on the leveling results acquisition time.

## 3. Results

### 3.1. Deformation along the Diversion Canal

#### 3.1.1. Analysis Based on Ascending SAR Images

**Figure 6:**
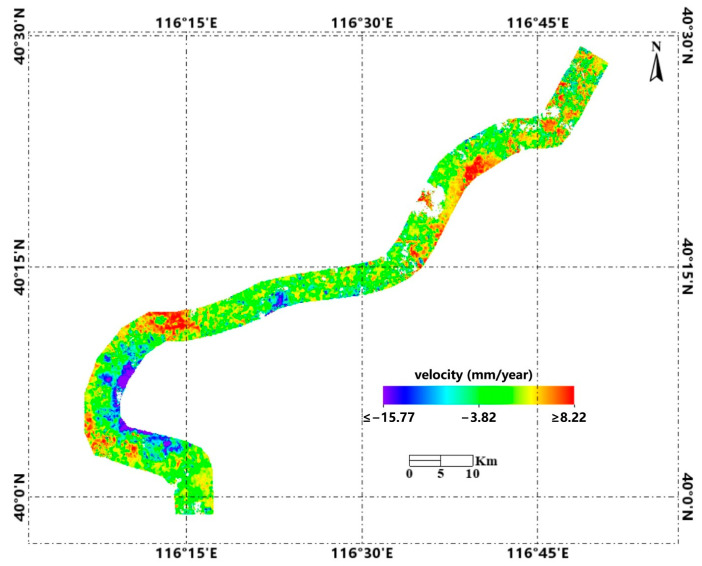
Subsidence velocity along the overall diversion canal from 2017 to 2023.

**Figure 7:**
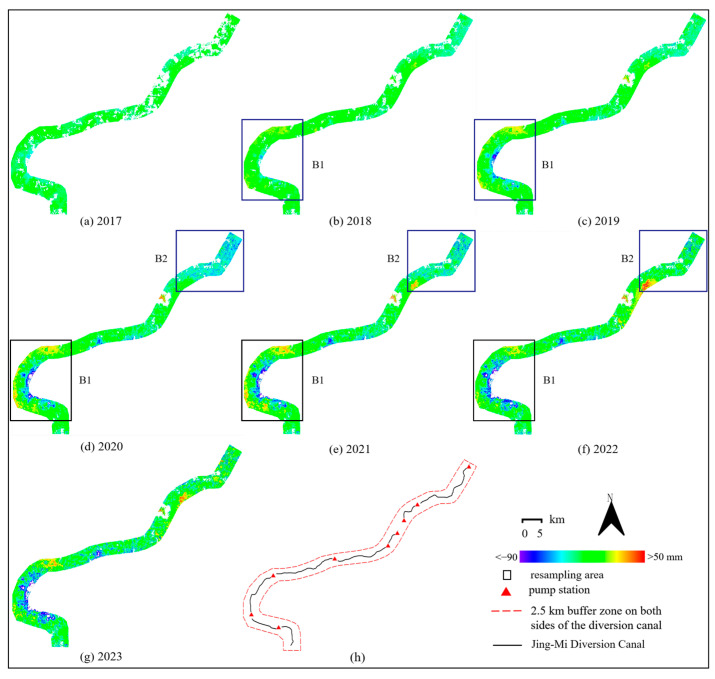
(**a**–**g**) Accumulated subsidence from 2017 to 2023. (**h**) The coverage diagram of buffer zone.

We used the monthly ascending SAR images to obtain accumulated subsidence along the entire diversion canal and discovered some risk areas. We drew an InSAR velocity map (Figure 6) to show the overall displacement pattern. From a global perspective, a general trend of subsidence along the diversion canal was observed, as shown in Figure 7. B1 and B2 are the resampling areas. B1 is from Tundian to Niantou pumping station, B2 is from Yanqi to Xiwengzhuang pumping station. The canal from Tundian pumping station to Niantou pumping station uplifted in 2018. It had been sinking from 2019 to 2022, until it slowed down in 2023. There was subsidence along the canal from Yanqi pumping station to Xiwengzhuang pumping station from 2020 to 2022. Specifically, there was uplift deformation around the Yanqi pumping station in 2021 and 2022. 

**Figure 8:**
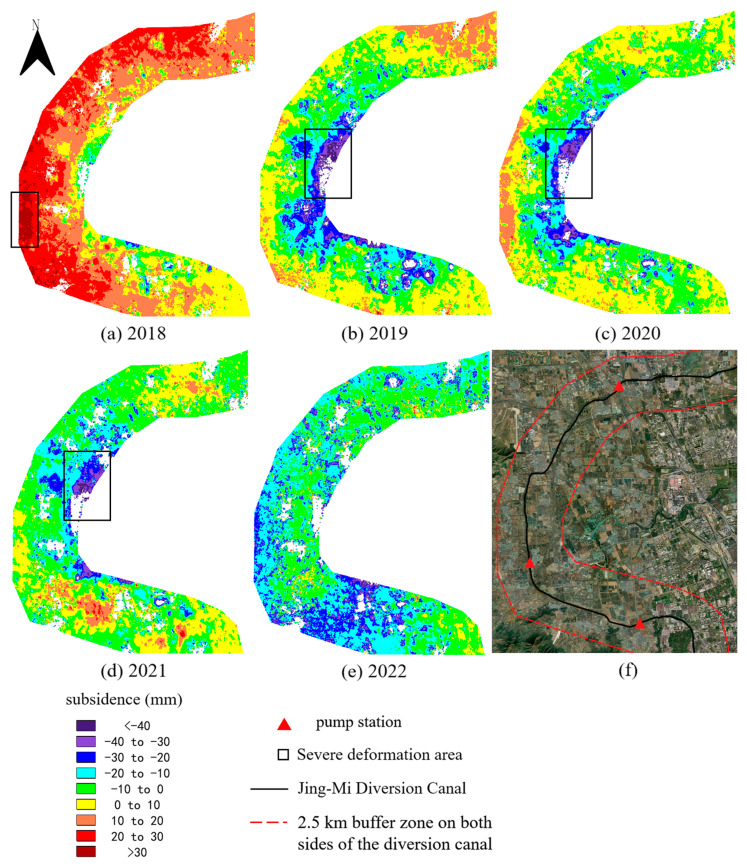
(**a**–**e**) Subsidence of resampled area B1 obtained by ascending SAR images. (**f**) The ESRI satellite imagery along the canal from Tundian to Niantou.

The results obtained from the above InSAR were limited by resolution and cannot reflect local deformation within a small area within a single year, making it impossible to conduct a more detailed assessment of the risk area. All SAR images were used to resample the two risk areas (B1 and B2) in Figure 7. We obtained resampling results of the ascending SAR images from Tundian to Niantou, as shown in Figure 8. This displays the deformation patterns of each year (each subgraph starts at the beginning of the year and ends at the end of the year). There was a large-scale uplift along the route in 2018, accounting for about 93%. The most severe region covered an area of approximately 3.48 km^2^. The average uplift deformation was 30.98 mm, and the maximum deformation reached 38.37 mm. The deformation patterns along the route from 2019 to 2021 were similar, showing uplift in the west and subsidence in the east. The area with the most severe subsidence in 2019 covered an area of approximately 1.72 km^2^, with an average subsidence of 44.68 mm and a maximum amount of 48.05 mm. In 2021, the area of this region decreased by about 23%, the average subsidence amount decreased by about 8%, and the maximum subsidence amount decreased by about 17%. At the same time, the uplifted areas along the route shifted toward the center and the total uplifted area accounted for about 33%. The subsidence situation was most severe in 2022, with sinking areas accounting for about 94% and an average subsidence of −12.75 mm.

**Figure 9:**
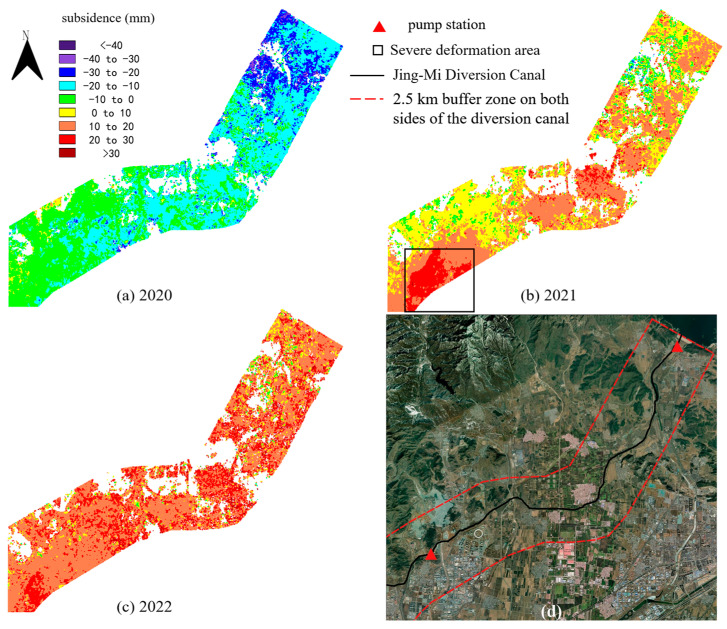
(**a**–**c**) Subsidence of resampled area B2 obtained by ascending SAR images. (**d**) The ESRI satellite imagery along the canal from Yanqi to Xiwengzhuang.

We obtained resampling results of the ascending SAR images from Yanqi to Xiwengzhuang, as shown in Figure 9. In 2020, 97% of the entire canal was sinking, with an average subsidence of 16.52 mm. In 2021, 92% of the area was uplifted. The largest uplift area was located on the south side of the Yanqi pumping station, with an area of approximately 5.58 km^2^. The average uplift deformation was 21.64 mm, and the maximum uplift deformation reached 34.40 mm. In 2022, 98% of the area along the route from Yanqi to Xiwengzhuang was uplifted, with an average vertical deformation of 17.65 mm along the entire route.

#### 3.1.2. Supplement Based on Descending SAR Images

**Figure 10:**
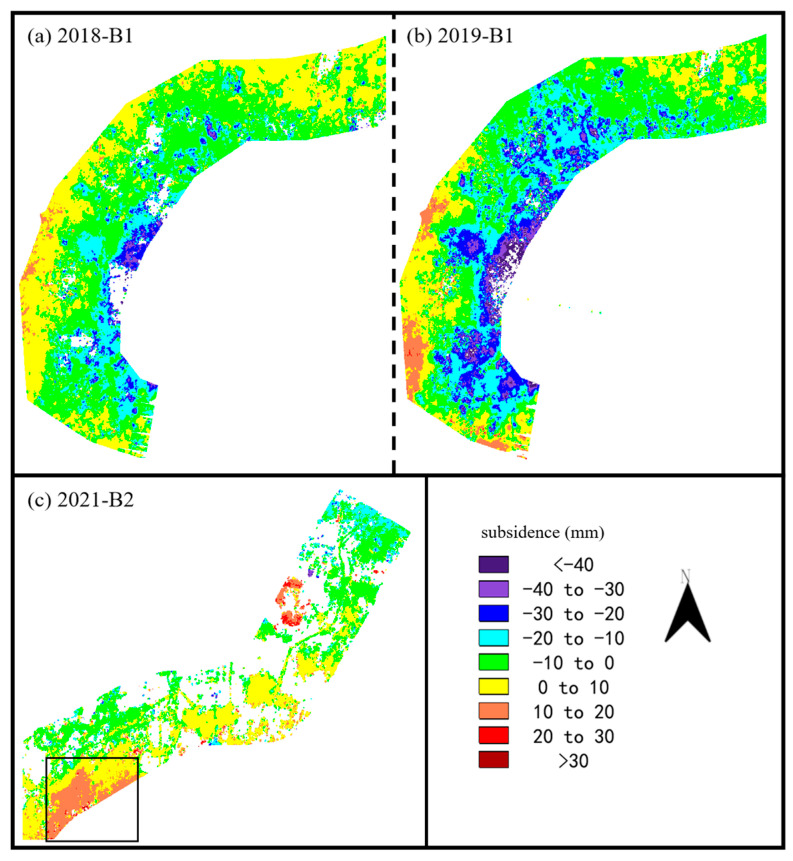
Subsidence obtained by descending SAR images. (**a**,**b**) Resampled area B1. (**c**) Resampled area B2.

In order to perform a more comprehensive analysis, we used descending SAR images to resample some areas. Due to the limited number of descending images in 2017 and 2020, the results of 2018, 2019 and 2021 were used for analysis. The subsidence along the canal from Tuntian to Niantou and Yanqi to Xiwengzhuang were extracted, as shown in Figure 10. In 2018, the eastern region from Tuntian to Niantou experienced subsidence, while the western region experienced uplift. In 2019, the deformation distribution was consistent with the observation of ascending images along the Tuntian to Niantou. In 2021, uplift deformation was also observed around the Yanqi pumping station.

**Figure 11:**
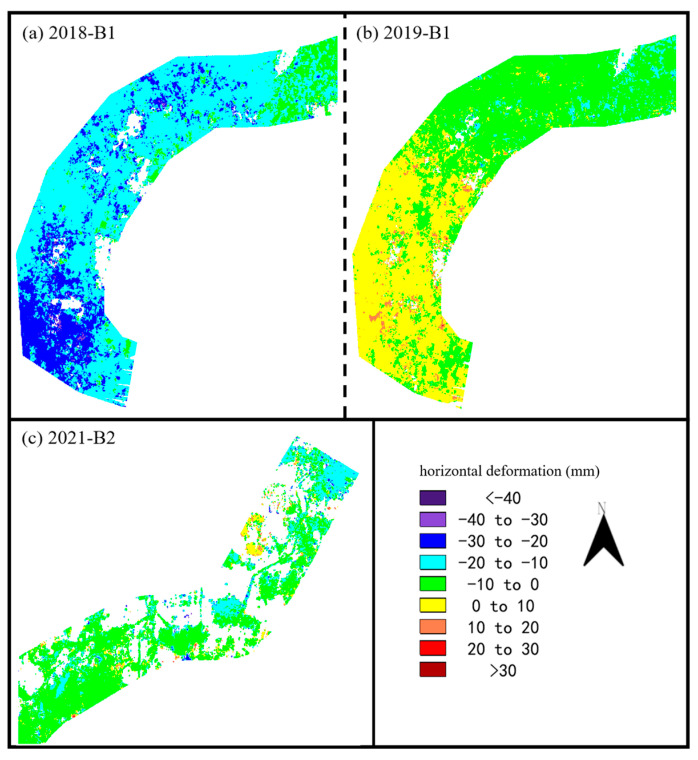
Horizontal deformation (east–west) obtained by ascending and descending SAR images. (**a**,**b**) Resampled area B1. (**c**) Resampled area B2.

According to Equation (3), we ignored the north–south deformation and solved for the east–west deformation by combining the results of the ascending and descending SAR images, as shown in Figure 11. In 2018, a westward deformation was observed along the Tundian to Niantou, with deformation values ranging from 0 to 30 mm. In 2019, there was 0–20 mm deformation to the east and west along the southern and northern sides of the Tundian to Niantou route, respectively. In 2021, about 91% of the area along the Yanqi to Xiwengzhuang route experienced westward deformation, with an overall average westward deformation of 6.69 mm.

### 3.2. Impact of Groundwater Level on Subsidence

**Figure 12:**
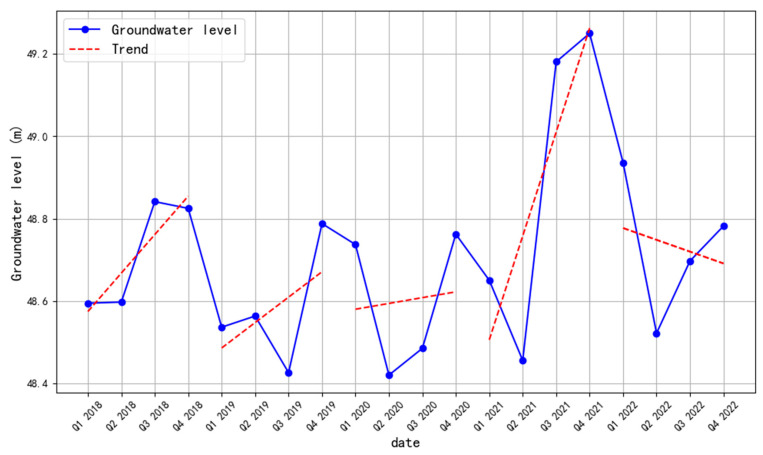
Groundwater level along the Tundian to Niantou from 2018 to 2022.

Some researchers have found that a decrease in groundwater level can lead to surface subsidence and deformation [39,40,41]. Figure 12 shows the quarterly average groundwater level changes along the Tundian to Niantou from 2018 to 2022. To explore the relationship between groundwater level changes and subsidence, we analyzed groundwater level changes within each year separately. It is clear that there was some uplift phenomenon along the canal from 2018 to 2021, and that the linear trend of groundwater level was upward in the first three quarters of each year. This may be due to the replacement of some groundwater extraction by water diversion projects [42]. In 2022, the linear trend of groundwater level decreases, with 94% of the areas along the route experiencing subsidence. To some extent, this phenomenon indicates a correlation between groundwater level and land subsidence. The rise of water level will exacerbate the ground uplift, while the fall of water level will exacerbate the ground subsidence. This view is consistent with some existing research [43,44,45].

**Figure 13:**
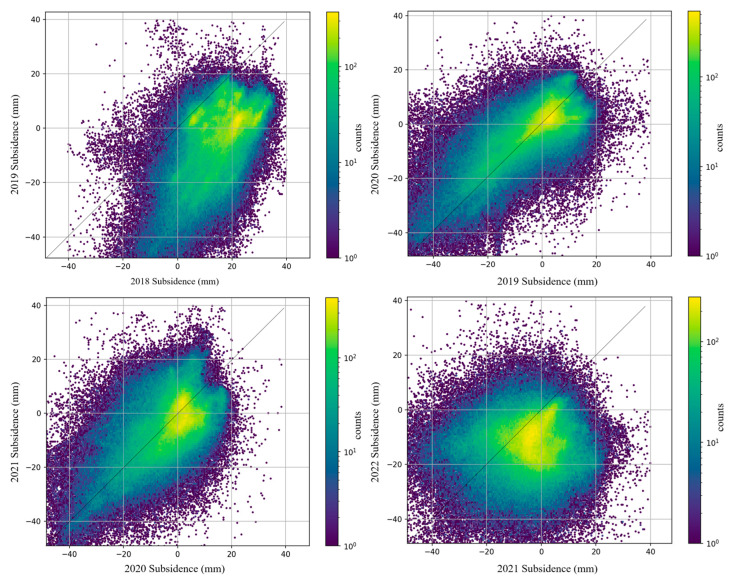
Pixel-by-pixel comparison of subsidence along the Tundian to Niantou from 2018 to 2022.

Next, a pixel-by-pixel comparison was performed on the distribution of the subsidence. We obtained the pixel-by-pixel comparison results of the Tundian to Niantou from 2018 to 2022, as shown in Figure 13. The subsidence results of 2019 and 2020 were evenly and symmetrically distributed on both sides of a straight line with a slope of 1, indicating similar subsidence conditions in the two years. In fact, the groundwater levels in these two years were close and had a consistent trend. Compared with 2020, the water level rise in 2021 increased by approximately 0.7 m. Of this area, a proportion of 7.3% experienced intensified uplift deformation. There were more pixels distributed between 20 and 40 mm in 2021. In the comparison of results between 2021 and 2022, the yellow core area was tilted towards 2021. This indicates that the subsidence situation was more severe in 2022, which is consistent with the changes in water level. The uplift phenomenon proportion reached 93% in 2018, but the water level rose only about 0.2 m. This may be due to lower water levels in previous years. The above results indicate some correlation between surface deformation and water level changes. Unfortunately, monitoring data along the route for 2017 and earlier were missing. Regular maintenance of the monitoring instruments should be intensified in the future.

### 3.3. Fine Monitoring of Pumping Station Buildings

The normal operation of pumping stations is related to the safety of water diversion projects. We used InSAR to monitor pumping stations in areas with severe subsidence. We verified the deformation detected by InSAR using leveling results. Taking Tundian, Qianliulin, and Niantou pumping stations as examples, we analyzed the results of InSAR and leveling measurement.

**Figure 14:**
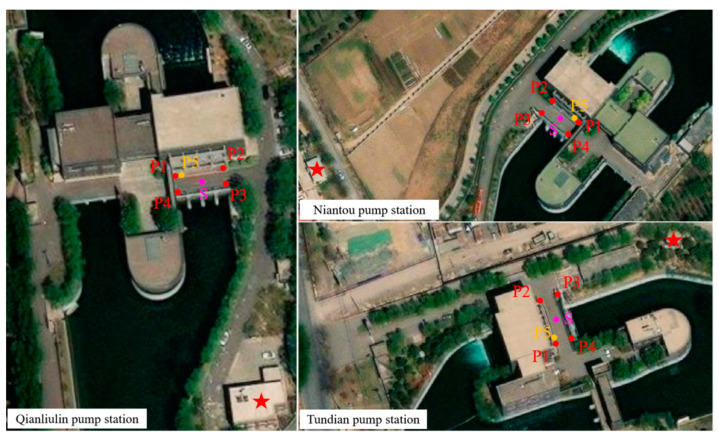
The ESRI satellite images of pumping stations. P1 to P4 are vertical monitoring points, P5 is horizontal monitoring point. S is the point for extracting InSAR deformation. Red star is reference point.

The positions of the three pumping stations are shown in Figure 1b. The subsidence observation of the main buildings began in December 2015. As shown in the remote sensing satellite image (Figure 14), there are four vertical displacement observation points (P1–P4) and one horizontal deformation observation point (P5) locate on the traffic bridge near the main pumping station. We have extracted the results from 20 May 2017 to 30 September 2023 for analysis. The foundation form of the traffic bridge is a box culvert. S is the point for extracting InSAR deformation. Surrounding stable bedrock or buildings are selected as reference points (red star symbol). For leveling measurement, the measured values of four vertical displacement observation points were averaged as the subsidence of the pumping station traffic bridge.

**Figure 15:**
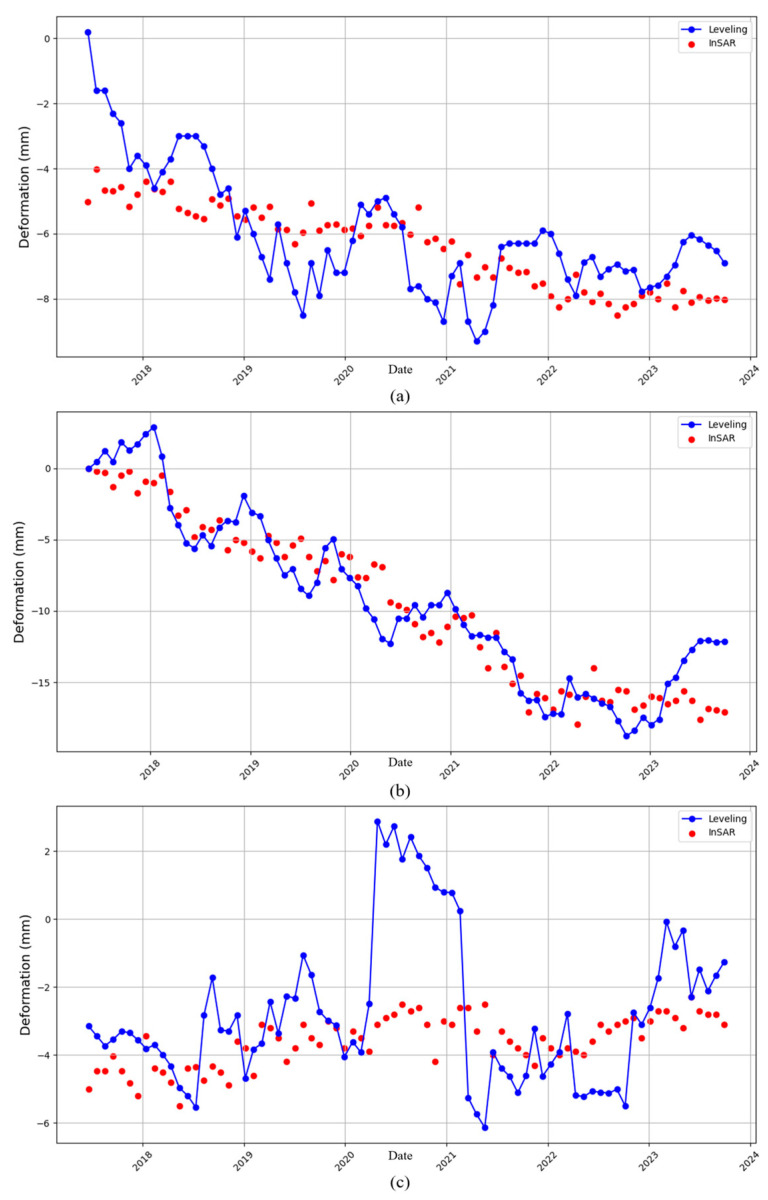
Comparison between InSAR results at point S and level measurement results. (**a**) Tundian pump station. (**b**) Qianliulin pump station. (**c**) Niantou pump station.

The subsidence and horizontal deformation values measured by leveling were projected to the LOS direction according to Equation (3). The original InSAR measurement values were directly adopted. The results are shown in Figure 15. The results obtained by leveling and InSAR show similar trends. The differences between InSAR and leveling results were calculated. The maximum error was within 10 mm, and the mean error and standard deviation were within 2 mm. The summary of the results is shown in Table 1. From various indicators, the accuracy reached the mm level. We suggest using this method for accuracy verification when SAR images are insufficient and when leveling data are sufficient.

## 4. Discussion

In addition to the groundwater level discussed in this article, there are many other factors that affect subsidence. For example, the construction of high-rise buildings [46,47] and vibrations caused by subway operations [48]. All of these have stressed the city and exacerbated subsidence. The periodic fluctuation of groundwater level is the direct cause of seasonal deformation, while rainfall is the main cause. In spring and summer, there is heavy rainfall and rainwater infiltration replenishes the groundwater. The increase in soil moisture strengthens the bedrock, thereby alleviating subsidence [49]. In fall and winter, there is less rainfall and groundwater support is lost, thereby intensifying subsidence [50]. In the future, it is necessary to conduct research on subsidence in different regions, identify the factors that have the greatest impact on subsidence, and explore the mechanisms of subsidence. Doing so will be conducive to ensuring the healthy development of cities.

In terms of accuracy verification, there is still a certain gap in the accuracy of InSAR when compared with the actual engineering requirement of 2 mm. However, the results obtained by InSAR are similar in trend to leveling measurements and can be used as a reference for relevant departments. At present, some researchers have attempted to extract and synthesize deformation using multi-source satellite data [51,52,53]. However, there are still difficulties, such as complex data acquisition processes, unequal timelines, and inconsistent coverage. In order to ensure the safety of water diversion, more satellite data should be adopted in the future to extract deformation from multiple directions. Such data could be acquired, for example, from small commercial satellites, such as HISEA-1 [54] and Chaohu-1 [55], that have shorter revisit cycles and higher resolution. Secondly, building a multi-source satellite dataset platform is beneficial for data acquisition. Finally, corner reflectors can be added to assist interferometric measurements in some channels with low coherence [56].

## 5. Conclusions

In this article, we used Sentinel-1 data to analyze the deformation along the Jing-Mi Diversion Canal based on SBAS InSAR and adopted a process from global to local. The conclusions are as follows:

1. Overall, monthly ascending SAR images were used to obtain deformation information. In the past seven years, the Jing-Mi Diversion Canal has been mainly characterized by subsidence and occasional uplift. Two areas with severe deformation were identified as the focus of investigation. The subsidence along the canal from Tundian to Niantou was the most severe, followed by Yanqi to Xiwengzhuang. The central area of the diversion canal was relatively stable. Subsequently, the monitoring of areas with severe deformation should be strengthened, including increasing the monitoring frequency and adding monitoring instruments.
2. Locally, resampling was performed on the key inspection areas, and calculations were performed using all images. The leveling measurement results of pumping stations are consistent with the trend of InSAR observation result and the error is within 10 mm. In general, InSAR measurement can provide a large-scale understanding of the deformation distribution of the water diversion canal. There was a certain correlation between the subsidence and groundwater level along the canal from Tundian to Niantou. The pixel-by-pixel comparison of the subsidence was consistent with the groundwater level hydrograph. The rise of groundwater level along the diversion canal will exacerbate ground uplift, while the fall of groundwater level will exacerbate ground subsidence.
3. Compared with other studies, we have supplemented and strengthened the verification of deformation accuracy and exploration of groundwater. Due to limitations in spatiotemporal resolution, relying solely on Sentinel-1 is insufficient to achieve comprehensive and precise monitoring of hydraulic engineering projects. More high-resolution images from small satellites, drones, etc. can be obtained to achieve data fusion. At the same time, it is necessary to develop new processing algorithms to timely process images and output deformation information, which is of great significance for ensuring engineering safety.

## Figures and Tables

**Table 1:** Summary of the differences between InSAR and leveling results.

Error Indicators (mm)	Maximum Error	Mean Error	Standard Deviation
Tundian pump station	5.226	1.278	0.867
Qianliulin pump station	5.062	1.773	1.185
Niantou pump station	5.978	1.652	1.449

## Data Availability

Sentinel-1 SAR images and 12.5m ALOS DEM can be obtained from the Alaska Satellite Facility for free, https://search.asf.alaska.edu (accessed on 22 November 2023).
